# ERK/NF-kB/COX-2 Signaling Pathway Plays a Key Role in Curcumin Protection against Acetaminophen-Induced Liver Injury

**DOI:** 10.3390/life13112150

**Published:** 2023-10-31

**Authors:** An-Hsun Chou, Hung-Chen Lee, Chia-Chih Liao, Huang-Ping Yu, Fu-Chao Liu

**Affiliations:** 1 Department of Anesthesiology, Chang Gung Memorial Hospital, Linkou Branch, Taoyuan 333, Taiwan; f5455@cgmh.org.tw (A.-H.C.); m7079@cgmh.org.tw (H.-C.L.); m7147@cgmh.org.tw (C.-C.L.); yuhp2001@cgmh.org.tw (H.-P.Y.); 2 College of Medicine, Chang Gung University, Taoyuan 333, Taiwan

**Keywords:** curcumin, acetaminophen-induced hepatotoxicity, inflammation, ERK/NF-kB/COX-2

## Abstract

Recent experimental studies have highlighted the beneficial effects of curcumin on liver injury induced by acetaminophen (APAP). However, the specific molecular mechanisms underlying curcumin’s hepatoprotective effects against APAP-induced liver injury remain to be fully elucidated. This study aimed to investigate the therapeutic effect of curcumin on APAP-induced liver injury using a mouse model. In the experiment, mice were subjected to an intraperitoneal hepatotoxic dose of APAP (300 mg/kg) to induce hepatotoxicity. After 30 min of APAP administration, the mice were treated with different concentrations of curcumin (0, 10, 25, or 50 mg/kg). After 16 h, mice with hepatotoxicity showed elevated levels of serum alanine transaminase (ALT), aspartate transaminase (AST), hepatic myeloperoxidase (MPO), TNF-α, and IL-6, and decreased levels of glutathione (GSH). Moreover, there was an increased infiltration of neutrophils and macrophages following intraperitoneal injection of APAP. However, curcumin-treated mice displayed a pronounced reduction in serum ALT, AST, hepatic MPO, TNF-α, and IL-6 levels, coupled with a notable elevation in GSH levels compared to the APAP-treated hepatotoxic mice. Moreover, curcumin treatment led to reduced infiltration of neutrophils and macrophages. Additionally, curcumin inhibited the phosphorylation of ERK and NF-kB proteins while reducing the expression of cyclooxygenase-2 (COX-2). These findings highlight the hepatoprotective potential of curcumin against APAP-induced liver injury through the suppression of the ERK, NF-kB, and COX-2 signaling pathways.

## 1. Introduction

Acetaminophen (N-acetyl-p-aminophenol; APAP) is a widely utilized pain-relieving and fever-reducing drug, and, when administered at appropriate therapeutic levels, typically considered safe. However, an overconsumption of acetaminophen can have severe consequences, leading to liver failure and death [1,2,3]. The mechanism underlying liver injury caused by acetaminophen involves the creation of a harmful metabolite known as N-acetyl-p-benzoquinone imine (NAPQI). This toxic metabolite is produced during the metabolism of acetaminophen in the liver, primarily by the cytochrome P450. Under normal circumstances, the liver’s antioxidant defense system, mainly reliant on glutathione (GSH), efficiently neutralizes NAPQI. Yet, in cases of APAP overdose, the production of NAPQI overwhelms the liver’s antioxidant capacity, leading to its accumulation and the subsequent depletion of GSH [4,5]. As the levels of GSH decrease, an excess of NAPQI forms covalent bonds with cellular proteins resulting in mitochondrial malfunction, reactive oxygen species (ROS) production, and DNA damage. These adverse effects can cause liver cell damage and provoke an inflammatory response [6,7,8,9].

Previous research has demonstrated the involvement of the liver’s inherent immune reaction in the advancement and gravity of hepatotoxicity. After initial damage to hepatocytes by APAP, resident liver macrophages called Kupffer cells (KCs) are activated by damage-associated molecular pattern (DAMP) molecules. Inflammatory agents such as chemotactic factors, cytokines, and reactive oxygen species (ROS) discharged by activated KCs will lure a greater number of immune cells, including invading macrophages and neutrophils, into the liver’s blood vessels, intensifying liver harm [10,11]. Recent study had underscored the participation of the extracellular signal-regulated kinase (ERK) signaling pathway in the onset of liver damage caused by APAP [12]. In the realm of traditional Chinese medicine, baicalin, in particular, had exhibited potential in mitigating liver injury induced by APAP through the inhibition of the ERK signaling pathway [12]. The ERK pathway is recognized for its role in controlling oxidative stress and inflammation, as well as in mediating mitochondrial dysfunction and apoptosis in conditions such as cisplatin-triggered renal damage [13]. Additionally, suppressing the ERK signaling pathway has been shown to lessen the extent of liver damage induced by APAP [14].

Currently, N-acetylcysteine (NAC) stands as the primary antidote for emergency treatment of acetaminophen (APAP) overdose. It works by reducing APAP-induced liver injury through enhanced glutathione synthesis. This process aids in detoxifying NAPQI and scavenging free radicals. However, its usage is linked to several side effects like vomiting, diarrhea, and anaphylactic reactions [15]. Despite being the standard clinical drug for addressing APAP overdose in emergencies, NAC’s efficacy is balanced against potential side effects. Hence, exploring alternative antidotes is crucial for effective emergency treatment of APAP-induced liver injury. Recently, there has been a worldwide burgeoning interest in the utilization of natural herbal products believed to promote a healthy lifestyle. Among these products, curcumin, which is sourced from the roots of Curcuma longa, and is more commonly recognized as turmeric, has gained widespread recognition for its therapeutic effectiveness, and it is generally considered to possess a favorable safety profile. As a result, it has become immensely popular for various health-related purposes [16]. Curcumin boasts a diverse range of biological and pharmacological activities. It has the capacity to combat inflammation, infections, and cancer [17]. Among the areas of research, particular attention has been drawn to its potential impact on liver injury. In cases of ethanol-induced liver injury, curcumin’s remarkable antioxidant and anti-inflammatory properties have displayed promise in countering oxidative stress and inflammation, thereby alleviating liver damage [18]. Similarly, curcumin has demonstrated its hepatoprotective effects against liver injury by iron overload [19]. In these scenarios, curcumin’s antioxidant activity and its ability to modulate inflammatory pathways play pivotal roles [20,21].

Curcumin has been shown to exhibit remarkable inhibitory properties against key inflammation mediators, including NF-kB, cyclooxygenase-2 (COX-2), lipoxygenase (LOX) enzymes (responsible for generating leukotrienes), and inducible nitric oxide synthase (iNOS) [22]. Remarkably, it has demonstrated significant preventive and therapeutic effects in diverse experimental animal models, spanning conditions such as lung injury, renal injury, and colitis [23,24,25]. Moreover, curcumin has demonstrated its potential in partially reversing fibrosis caused by CCl4. Its effectiveness in both preventing and reversing cirrhosis appears to be associated with its ability to decrease the expression of TGF-β [26]. These findings underscore curcumin’s potential as an effective treatment for hepatic diseases. Furthermore, within the realm of hepatotoxicity, there is currently no knowledge regarding the engagement of the ERK/NF-kB signaling pathway in regulating COX-2 expression and the potential for COX-2-mediated production of pro-inflammatory cytokines in macrophages. Prior animal studies have underscored the positive impact of curcumin in ameliorating liver injury triggered by APAP. These investigations evaluated organ function and delved into the involvement of CYP2E1 and the Keap1-Nrf2 pathway concerning APAP-induced liver injury linked to oxidative stress [27,28]. However, the specific molecular mechanisms underpinning curcumin’s anti-inflammatory and hepatoprotective effects against APAP-induced toxicity were not delineated in these studies. In addition to oxidative stress, inflammation plays a pivotal role in the progression of APAP-induced liver injury, presenting a critical target for mitigating liver damage [12]. Although previous studies have explored the antioxidant effects of curcumin in the context of APAP-induced liver injury, the specific molecular mechanisms underlying curcumin’s anti-inflammatory hepatoprotective effects against APAP-induced liver injury have not been thoroughly addressed in prior research. Therefore, our study aimed to make a valuable contribution by delving into an alternative anti-inflammatory signaling pathway by utilizing a mouse model that simulates APAP-induced liver injury. Through this research, we hoped to gain a deeper understanding of curcumin’s role in safeguarding the liver from APAP-induced liver injury, and to contribute to a broader comprehension of its hepatoprotective properties.

## 2. Materials and Methods

### 2.1. Animals and Experimental Model and Treatment

We employed adult male C57BL/6 (B6) mice, purchased from BioLASCO Taiwan Co., Ltd. (Taipei, Taiwan), for this study. All procedures were conducted according to the guidelines of the Animal Welfare Act and the Guide for the Care and Use of Laboratory Animals by the National Institute of Health. 

Specifically, male mice aged 8 to 10 weeks from the experimental group were employed, showcasing body weights ranging from 15 to 20 g. To establish the experimental conditions, the mice underwent intraperitoneal (IP) injection of APAP at a dose of 300 mg/kg to induce acute liver injury [12,29]. The mice were allocated randomly into six groups, each group comprising six mice. Four of the groups were administered an intraperitoneal injection of APAP (300 mg/kg) dissolved in sterile phosphate-buffered saline (PBS). Subsequently, they were given intraperitoneal injections of curcumin at varying doses: 0 mg/kg, 10 mg/kg, 25 mg/kg, or 50 mg/kg, 30 min after APAP injection. In the two remaining groups, mice received either PBS (representing the control group) or curcumin at a dosage of 50 mg/kg. Following a 16 h experimental period, all groups of animals were sacrificed by cervical dislocation under isoflurane anesthesia. Blood and liver samples were obtained for further analysis.

### 2.2. Liver Function Assessment

To assess the possible hepatotoxicity, we conducted an evaluation of liver functions. This involved the collection of blood samples, the subsequent separation of serum, and the measurement of the AST and ALT levels in liver. These measurements were performed using the Vitros DT60 II Chemistry System (Ortho-Clinical Diagnostics; Johnson & Johnson, New York, NY, USA).

### 2.3. GSH Assessment in Liver 

The GSH level was measured by glutathione assay kit (Cayman chemical Company, Ann Arbor, MI, USA) with absorbance readings at 412 nm by spectrophotometer and the GSH level was reported as μmol/g tissue [30]. 

### 2.4. Liver MPO Activity Assay

A 96-well microtiter plate was used for sample processing and analysis. Liver tissues were collected, and they were then submerged in a cold buffer to maintain sample integrity. Subsequently, the tissues were homogenized to ensure even enzyme distribution. Homogenized liver-tissue samples were subjected to centrifugation, separating cellular debris from the liquid supernatant. MPO, an inflammation marker, was quantified spectrophotometrically using a reaction mixture composed of o-dianisidine (10 mg/mL) and 0.3% hydrogen peroxide (H_2_O_2_). To ensure the reliability of results, the assay was conducted in triplicate for each liver-tissue sample. The maximum absorbance at 460 nm was utilized to accurately calculate MPO activity in units specific to the assay [31]. 

### 2.5. Histology

Liver tissues were harvested and then fixed in a solution of 4% paraformaldehyde at room temperature for 30 min. Paraformaldehyde is a common fixative used to preserve tissue structures. After fixation, the tissues were embedded in paraffin wax. The paraffin-embedded liver tissues were subsequently moved to 70% ethanol. The paraffin blocks containing the tissue samples were sliced into thin sections (4 μm). And then, selected sections were stained using hematoxylin and eosin (H&E) allowing for the visualization of tissue architecture and the assessment of cellular details. The proportion of necrotic tissue in liver sections was assessed by analyzing the count of microscopic fields showing necrosis in proportion to the complete histological section. The study was executed by two investigators who maintained blinding throughout the process to eliminate potential bias in the interpretation of results.

For immunohistochemical staining, sections underwent a series of steps: deparaffinization in xylene to remove paraffin, rehydration in alcohol and distilled water, antigen retrieval to enhance antibody binding, and neutralization of endogenous peroxidase activity using a three percent H_2_O_2_ solution. This was followed by multiple washes with TBS-Triton and TBS buffers to prepare the tissues for antibody incubation. For LYG6 and Mac-2 immunohistochemical staining, tissue sections were incubated with primary rat anti-mouse antibody at a concentration of 1 ug/mL, followed by the polymer-conjugated anti-rat antibody (Histofine Simple Stain Mouse MAX PO (Rat), Nichirei, Tokyo, Japan) and washed with TBS [32]. For the substrate–chromogen reaction, diaminobenzidine tetrahydrochloride (Nichirei) was used according to the manufacturer’s protocol. Mounted preparations were examined under a light microscope.

### 2.6. Quantifying Cytokine Levels in Mouse Liver

After homogenizing the liver tissues, the supernatants were carefully collected. The total protein concentration was assessed by fluorescamine protein assay. After adjusting the samples for protein concentration, we utilized an eBiosciences ELISA kit (San Diego, CA, USA) to analyze cytokine expression, adhering to the supplied protocol. In every experiment, standard curves were incorporated.

### 2.7. Western Blotting

After homogenizing the liver tissues using a protein-extraction solution, the resulting lysates underwent centrifugation at 12,000× *g* for 10 min at 4 °C, repeated twice for clearance. Protein concentrations were assessed using the Bradford protein microassay. Each sample was loaded with the same protein concentration and underwent electrophoresis on 10% sodium dodecyl sulfate polyacrylamide gels. Following that, proteins were transferred from the gel onto polyvinylidene fluoride membranes. Blocking of the membranes was accomplished by incubating them in a 5% fat-free milk solution for 1 h. Following this, a sequence of three washes was executed utilizing Tris buffer with 1% Tween^®^ 20. The membrane was incubated in 1:1000 dilution of ERK, phospho-ERK (p-ERK), NK-kB, phospho-NF-kB, and COX-2 (Cell Signaling Technology, Danvers, MA, USA) primary antibodies overnight at a temperature of 4 °C. Subsequent to primary-antibody exposure, a secondary antibody tagged with horseradish peroxidase was introduced to the membranes and they were left at room temperature for a duration of 1 h. The proteins detected through immunodetection were visualized utilizing the enhanced chemiluminescence (ECL) system. For validation of consistent protein loading, the membranes underwent a stripping process followed by re-probing using a monoclonal antibody targeting β-actin. Quantification of gel bands was achieved through densitometry, and the normalization to β-actin was performed to ensure a precise comparison of protein levels.

### 2.8. Statistical Analysis 

All reported results are presented as the mean value ± the standard error of the mean (SEM). The sample size for each group is denoted as “n = 6 mice per group”. The statistical calculations were conducted using Prism version 5.0. To compare the experimental and control groups, unpaired *t*-tests were used for direct comparisons between two groups. One-way analysis of variance (ANOVA) was utilized for comparisons involving more than two groups. Post hoc Tukey multiple comparisons were performed to identify specific group differences. Statistical significance was set at a threshold of *p* < 0.05. Any result with a *p*-value less than 0.05 was considered statistically significant.

## 3. Results

### 3.1. Protective Effects of Curcumin against APAP-Induced Liver Injury

**Figure 1:**
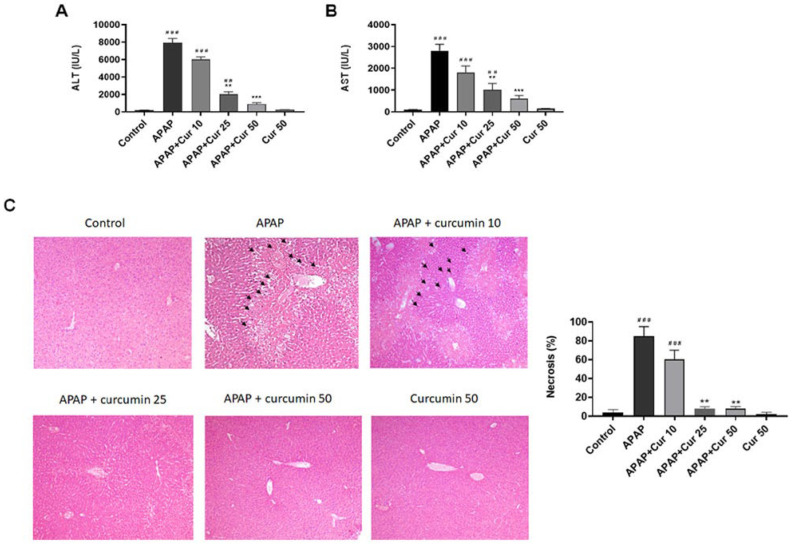
Effects of curcumin on APAP-induced liver injury. Serum ALT levels (**A**), AST levels (**B**), and histopathological changes with H&E staining (50×) (**C**) in acetaminophen-induced hepatic injury. Mice received injections of PBS (control), APAP at a 300 mg dose, or curcumin (10, 25, or 50 mg/kg) administered 30 min following the APAP injection. Additionally, a group received curcumin (50 mg/kg) alone. The mice were then euthanized after 16 h. Cell necrosis (black arrows) was evaluated and the percent of necrosis was estimated by evaluating the number of microscopic fields with necrosis compared to the entire histologic section. ## *p* < 0.01, ### *p* < 0.005 vs. control group; ** *p* < 0.01, *** *p* < 0.005 vs. APAP.

ALT and AST levels exhibited a significant increase (*p* < 0.005) in liver injury caused by APAP, whereas no discernible differences were noted between the PBS control and 50 mg/kg curcumin groups. However, treatment with 25 and 50 mg/kg curcumin resulted in a substantial reduction in serum ALT and AST levels. Conversely, treatment with 10 mg/kg curcumin did not produce a significant decrease in serum ALT and AST concentrations (Figure 1A,B).

Subsequently, we examined the impact of curcumin treatment on histopathological alterations following liver injury induced by APAP. H&E staining unveiled pronounced sinusoidal swelling, centrilobular necrosis, and central vein endothelium disruption in the APAP-induced liver injury group. Notably, the groups receiving curcumin at doses of 25 or 50 mg/kg subsequent to APAP administration exhibited effective attenuation of these pathological changes, displaying well-preserved hepatocytes with reduced necrosis and diminished sinusoidal swelling. In contrast, treatment with curcumin at a dosage of 10 mg/kg did not induce a significant change when compared to the APAP-only group (Figure 1C).

### 3.2. Effects of Curcumin on Hepatic GSH Expression Levels 

**Figure 2:**
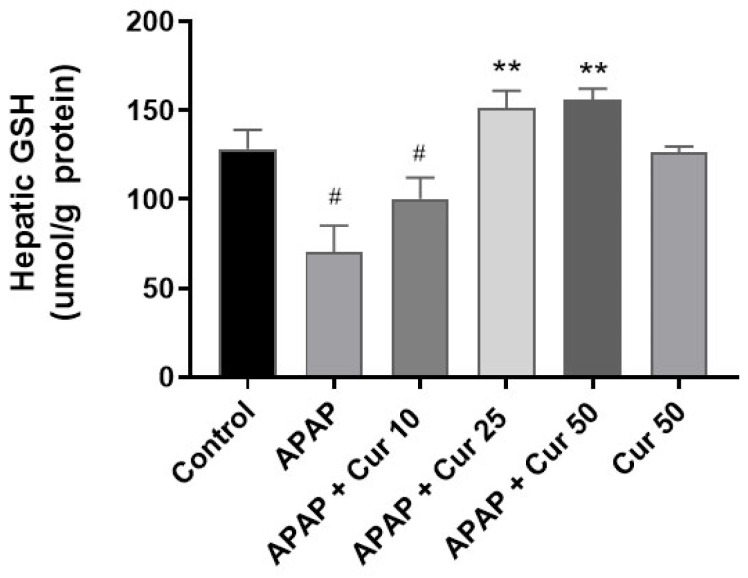
Effects of curcumin on hepatic GSH levels. Mice received injections of PBS (control), APAP at a 300 mg dose, or curcumin (10, 25, or 50 mg/kg) administered 30 min following the APAP injection. Additionally, a group received curcumin (50 mg/kg) alone. Afterward, the mice were sacrificed to assess hepatic expression GSH levels. ^#^ *p* < 0.05 vs. control; ** *p* < 0.01 vs. APAP.

Figure 2 shows the effects of curcumin on hepatic GSH levels. No significant differences were observed in hepatic GSH levels between the control group and the curcumin-only treatment groups. However, GSH levels were markedly reduced in the APAP-only group compared to the control group (*p* < 0.05). Importantly, curcumin treatment at doses of 25 or 50 mg/kg substantially restored hepatic GSH levels (*p* < 0.01). Hepatic GSH levels did not significantly differ following treatment with 10 mg/kg curcumin when compared to APAP. This information enriches the significance of our study by shedding light on potential biochemical mechanisms underlying curcumin-related liver protection.

### 3.3. Effects of Curcumin on MPO Activity and Neutrophil Accumulation in APAP-Induced Liver Injury

**Figure 3:**
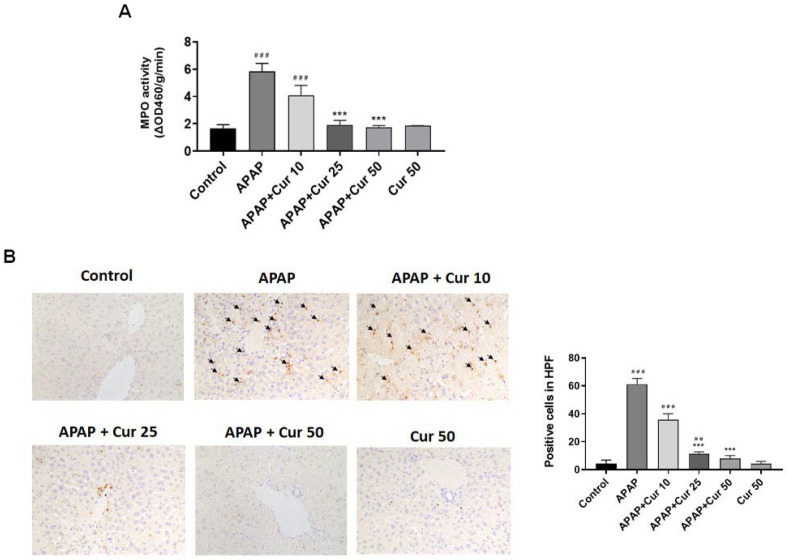
Effects of curcumin on hepatic MPO activity and neutrophil accumulation. (**A**) Mice received injections of PBS (control), APAP at a 300 mg dose, or curcumin (10, 25, or 50 mg/kg) administered 30 min following the APAP injection. Additionally, a group received curcumin (50 mg/kg) alone. The mice were then euthanized for the evaluation of MPO activity. ### *p* < 0.005 vs. control group; *** *p* < 0.005 vs. APAP group. (**B**) Mice received injections of PBS (control), APAP at a 300 mg dose, or curcumin (10, 25, or 50 mg/kg) administered 30 min following the APAP injection. Additionally, a group received curcumin (50 mg/kg) alone. The mice were then euthanized for the evaluation immunohistochemical staining. Liver sections were immunostained for neutrophils (black arrows, 200×). ## *p* < 0.01, ### *p* < 0.005 vs. control group; *** *p* < 0.005 vs. APAP.

Figure 3A depicts MPO activity of the liver. There was a significant increase in liver MPO activity in the APAP group compared to the control group (5.8 ± 0.3 vs. 1.6 ± 0.17 ΔOD460/g/min, *p* < 0.005). Administration of curcumin resulted in a reduction in liver MPO activity. The 25 mg/kg (1.8 ± 0.2 vs. 5.8 ± 0.3 ΔOD460/g/min, *p* < 0.005) and 50 mg/kg (1.76 ± 0.06 vs. 5.8 ± 0.3 ΔOD460/g/min, *p* < 0.005) curcumin groups showed a substantial reduction in MPO activity compared to the APAP group. However, hepatic MPO activity did not significantly decrease following treatment with 10 mg/kg curcumin when compared to APAP (Figure 3A).

Mice treated solely with APAP displayed elevated neutrophil infiltration within the liver parenchyma, as indicated by LY6G, a marker specific to granulocytes, in comparison to the control mice. In contrast, the groups receiving curcumin at doses of 25 or 50 mg/kg subsequent to APAP administration exhibited a notable reduction in neutrophil infiltration within the liver parenchyma (Figure 3B).

### 3.4. Effects of Curcumin on Macrophages in APAP-Induced Liver Injury

**Figure 4:**
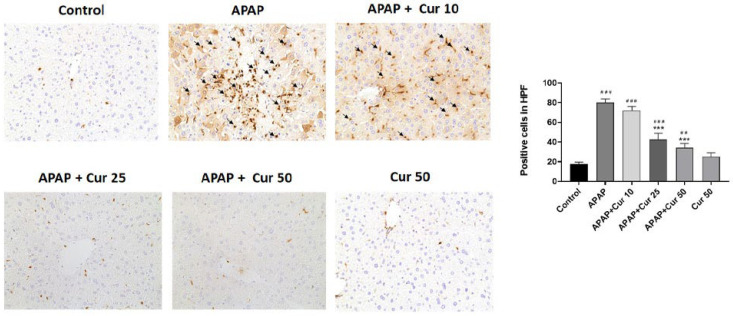
Effects of curcumin on macrophage accumulation. Mice received injections of PBS (control), APAP at a 300 mg dose, or curcumin (10, 25, or 50 mg/kg) administered 30 min following the APAP injection. Additionally, a group received curcumin (50 mg/kg) alone. The mice were then euthanized for the evaluation of immunostaining for macrophages (black arrow, 200×). Representative images were chosen from each group. ## *p* < 0.01, ### *p* < 0.005 vs. control group; *** *p* < 0.005 vs. APAP.

Mice treated only with APAP displayed higher macrophage infiltration within the liver parenchyma, as indicated by Mac2, a marker specific to macrophages, in comparison to the control mice. Nevertheless, administering curcumin at doses of 25 or 50 mg/kg post-APAP injection led to a notable reduction in the recruitment of macrophages around the hepatotoxic region (Figure 4).

### 3.5. Effects of Curcumin on Hepatic IL-6 and TNF-α Expression Levels

**Figure 5:**
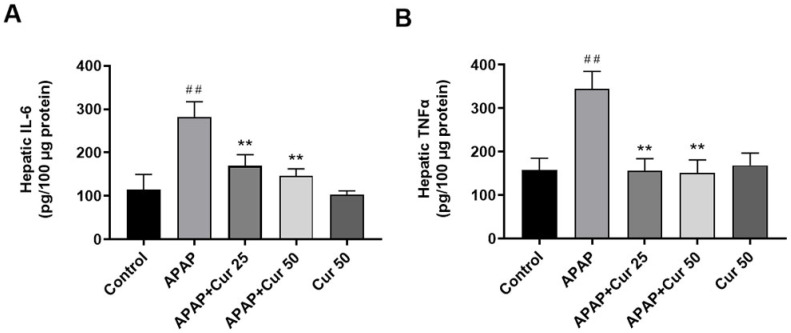
Effects of curcumin on hepatic IL-6 (**A**) and TNF-α (**B**) levels. Mice received injections of PBS (control), APAP at a 300 mg dose, or curcumin (25 or 50 mg/kg) administered 30 min following the APAP injection. Additionally, a group received curcumin (50 mg/kg) alone. Afterward, the mice were sacrificed to assess hepatic expression of IL-6 and TNF-α levels. ^##^ *p* < 0.01 vs. control; ** *p* < 0.01 vs. APAP.

No significant differences were observed in hepatic IL-6 and TNF-α levels between the control group and the curcumin-only treatment groups. However, IL-6 and TNF-α levels were markedly increased in the APAP-only group compared to the control group (*p* < 0.01). Significantly, curcumin treatment at doses of 25 or 50 mg/kg substantially reduced hepatic TNF-α and IL-6 levels (*p* < 0.01) (Figure 5). 

### 3.6. Effects of Curcumin on Hepatic ERK, NF-kB, and COX-2 Protein Expression and Activity

**Figure 6:**
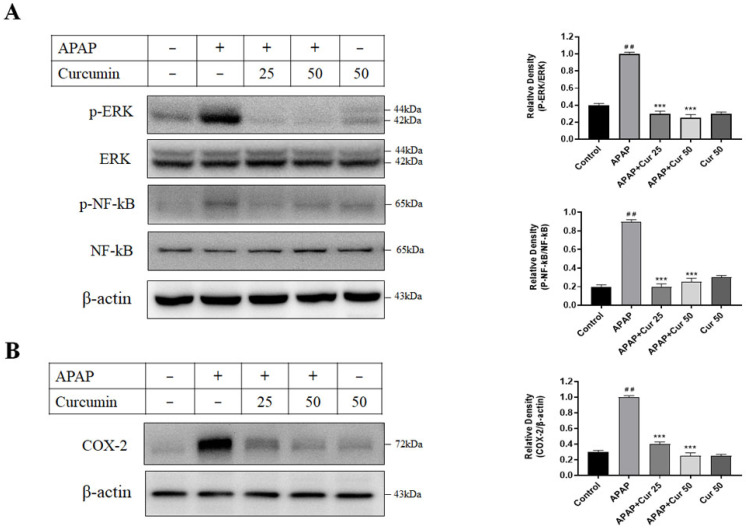
Effects of curcumin on hepatic ERK, NF-kB, and COX-2 protein expression and activity. (**A**) Protein expression of hepatic ERK, p-ERK, NF-kB, and p-NF-kB, and (**B**) protein expression of hepatic COX-2 from mice subjected to PBS (control), APAP at a 300 mg dose, or curcumin (25 or 50 mg/kg) administered 30 min following the APAP injection. Additionally, a group received curcumin (50 mg/kg) alone. Blots were subsequently probed for β-actin, ensuring uniform protein loading across all lanes. ## *p* < 0.01 vs. control; *** *p* < 0.005 vs. APAP.

Figure 6 displays the protein expressions of hepatic ERK, NF-kB, and COX-2. Following a single APAP dose, activities of ERK, NF-kB, and COX-2—determined by phosphorylation levels—markedly increased in the APAP group in comparison to the control group. Intriguingly, treatment with curcumin (at doses of 25 or 50 mg/kg) administered 30 min after APAP administration significantly reduced hepatic phosphorylated ERK, NF-kB, and COX-2 expression. 

## 4. Discussion

This study investigated the protective effects of curcumin on liver injury caused by APAP using a mouse model. Sixteen hours after APAP injection, hepatotoxic mice exhibited elevated concentrations of serum ALT, serum AST, liver MPO, TNF-α, and IL-6, and decreased levels of GSH. Moreover, there was an increased infiltration of neutrophils and macrophages following intraperitoneal injection of APAP. However, treatment with 25 or 50 mg/kg curcumin significantly improved hepatic injury. Curcumin-treated mice displayed a pronounced reduction in serum ALT, AST, hepatic MPO, TNF-α, and IL-6 levels, coupled with a notable elevation in GSH levels compared to the APAP-treated hepatotoxic mice, underscoring the protective effects of curcumin on the liver. Moreover, curcumin treatment led to reduced infiltration of neutrophils and macrophages. Additionally, curcumin inhibited the phosphorylation of ERK and NF-kB protein while reducing COX-2 expression levels. These results suggest that curcumin’s protective effects on APAP-induced hepatotoxicity are linked to anti-inflammatory mechanisms through the regulation of ERK/NF-kB/COX-2.

Curcumin, a natural compound derived from turmeric, offers hepatoprotective effects that encompass a reduction in liver inflammation, prevention of hepatocyte damage, and enhancement of overall liver function [27]. This multifaceted compound is well-regarded for its robust antioxidant and anti-inflammatory properties, which play a crucial role in combating oxidative stress and alleviating liver inflammation typically associated with liver diseases [33,34]. Furthermore, curcumin augments the activity of phase II detoxification enzymes, effectively aiding in the removal of toxins from the liver. Its ability to safeguard the liver against the toxic effects of various substances, including alcohol, drugs, and environmental toxins has also been demonstrated [35]. Moreover, curcumin has been found to stimulate the regeneration of liver cells, a vital process in repairing liver damage [26]. Additionally, it can lower the levels of pro-inflammatory cytokines such as TNF-α, which are frequently elevated in liver diseases [36]. Moreover, a study demonstrated that curcumin effectively mitigates obesity-induced liver-related complications by addressing multiple mechanisms, including weight reduction, lipid regulation, inflammation suppression, macrophage reduction, and the restoration of mitochondrial function, suggesting its potential in preventing and managing nonalcoholic fatty liver disease [37]. In light of these findings, further research is imperative to gain a comprehensive understanding of the mechanisms underlying curcumin’s anti-inflammatory effects in liver injury and its potential therapeutic applications, particularly in the context of APAP-induced liver injury.

In our research, the dramatic elevation in serum ALT and AST levels observed in the APAP-only group indicates severe liver damage, a hallmark of APAP toxicity. These findings are consistent with previous reports of APAP-induced hepatotoxicity [12]. However, the administration of curcumin at doses of 25 or 50 mg/kg resulted in a substantial reduction in serum ALT and AST levels. This indicates that curcumin effectively mitigates the liver injury caused by APAP. Notably, there were no discernible differences between the control group and the 50 mg/kg curcumin group, suggesting that curcumin at this dose level effectively preserves liver function. Histological examination revealed severe pathological changes in the livers of mice subjected to APAP, including sinusoidal swelling, necrosis at the centrilobular region, and disruption of the central vein endothelium. These findings are consistent with the characteristic features of APAP-induced liver injury [9]. Treatment with 25 or 50 mg/kg curcumin after APAP administration effectively mitigated these histological changes. This suggests that curcumin not only reduces biochemical markers of liver injury but also preserves liver tissue integrity. These protective effects are vital, as they can prevent the progression of liver damage and subsequent complications. GSH is a crucial endogenous antioxidant that plays a central role in protecting the liver from oxidative damage. The marked reduction in hepatic GSH levels observed in the APAP-only group is consistent with previous reports, as APAP metabolism depletes GSH, leading to increased susceptibility to oxidative stress [38]. Importantly, curcumin treatment at doses of 25 or 50 mg/kg substantially restored hepatic GSH levels. This is a critical finding as it sheds light on the potential biochemical mechanisms underlying curcumin-related liver protection.

The activation and infiltration of neutrophils and macrophages into the hepatic vasculature play pivotal roles in liver injury caused by APAP. Previous studies have highlighted the participation of neutrophils in liver damage during ischemia-reperfusion [39] and alcohol-induced hepatitis [40]. In the context of liver injury caused by APAP, previous research has demonstrated a strong correlation between disease progression and the increased accumulation of neutrophils [41]. This augmented neutrophil presence is linked to their infiltration into liver parenchyma, which is a process accompanied by the liberation of cytokines and chemokines from hepatocytes that have suffered damage [41]. Elevated tissue MPO levels are indicative of the presence of infiltrating neutrophils. Additionally, macrophages typically work in concert with neutrophils by producing pro-inflammatory cytokines. In our study, we confirmed that macrophages upregulated the expression of IL-6 and TNF-α in response to APAP-induced liver toxicity. It has been proposed that liver injury caused by APAP triggers sterile inflammation, and when cells undergo initial death, they release DAMP molecules, which then trigger the production of cytokines as a subsequent response. This inflammatory response results in the recruitment of neutrophils and monocytes into the liver blood vessels [10]. There was also evidence of macrophages derived from circulating monocytes that infiltrated the liver following APAP-induced liver injury [42]. Along with this results, LY6G and Mac-2, specific markers for granulocytes and macrophages, revealed significant reductions following the administration of 25 or 50 mg/kg curcumin after APAP treatment. These results clearly demonstrated that curcumin has protective effects against APAP-induced liver injury, which appear to be associated with an anti-inflammatory mechanism. IL-6 and TNF-α are pro-inflammatory cytokines that are elevated in various inflammatory conditions, including APAP-induced liver injury [12]. The marked increase in hepatic IL-6 and TNF-α levels in the APAP-only group underscores the presence of significant inflammation. Curcumin treatment at doses of 25 or 50 mg/kg substantially reduced hepatic TNF-α and IL-6 levels. This suggests that curcumin’s protective effects are associated with the suppression of pro-inflammatory cytokines, further contributing to its anti-inflammatory properties.

APAP overdose is known to induce severe liver damage characterized by inflammation [12]. Curcumin has exhibited the capacity to inhibit the production of inflammatory mediators, including prostaglandins and leukotrienes, by suppressing the activity of COX-2 and LOX enzymes [17]. Through this mechanism, curcumin effectively curtails the production of these inflammatory molecules, thereby mitigating the inflammatory cascade. Furthermore, by impeding NF-kB activation, curcumin can effectively reduce the expression of TNF-α, IL-1β, and IL-6, all of which play significant roles in the inflammatory process [43]. However, the specific anti-inflammatory effects of curcumin in the context of APAP-induced liver injury remain to be fully elucidated.

Two primary COX isoforms, COX-1 and COX-2, are encoded by distinct genes. COX-1 is consistently expressed in most tissues and plays a role in maintaining homeostatic balance. In contrast, inducible COX-2 is found in many immune cells, particularly macrophages, and is widely recognized for its pivotal role in inflammation. It accomplishes this by converting arachidonic acid into pro-inflammatory prostaglandins, predominantly prostaglandin E2 (PGE2), and triggering the production of other pro-inflammatory chemokines and cytokines [44]. The aberrant overexpression of COX-2 has been implicated in the development of various human cancers and inflammatory conditions [45]. As such, this inducible enzyme holds potential as a valuable surrogate biomarker for assessing the efficacy of chemopreventive strategies.

An accumulating body of research highlights the involvement of the ERK signaling pathway in inflammation [46,47]. In mouse models, the ERK pathway assumes a significant role in cisplatin-induced acute renal failure, primarily contributing to inflammation and apoptosis [48]. Inhibition of the ERK pathway has demonstrated potential in protecting against APAP-induced hepatotoxicity, notably by reducing the formation of reactive oxygen species (ROS) [48,49]. ERK signaling is pivotal in regulating the expressions of COX-2 and iNOS, thus playing a crucial role in both initiating and sustaining inflammatory responses [50]. Activated ERK can translocate into the nucleus, where it phosphorylates various transcription factors, including those involved in AP-1- and/or NF-kB-mediated gene expression. Subsequently, these transcription factors bind to the promoter region of the COX-2 gene, leading to an upregulation of COX-2 enzyme expression [51].

Inhibiting the activation of NF-kB has been associated with anti-inflammatory effects. A study observed that an NF-kB inhibitor suppressed the expression of COX-2, TNF-α, and IL-6 induced by cutaneous anaphylaxis, underscoring its role in mitigating inflammation [52]. Furthermore, NF-kB activity has been established as a key player in the regulation of COX-2 and iNOS expression [22]. Once COX-2 is expressed, it catalyzes the conversion of arachidonic acid into PGE2, which serves as a signaling molecule that further amplifies the inflammatory response. Notably, PGE2 can activate specific cell surface receptors, triggering signaling cascades that ultimately converge on the activation of NF-kB. This intricate cascade underscores the interconnectedness between ERK, NF-kB, and COX-2 in the context of inflammation [51].

Curcumin has demonstrated impressive inhibitory capabilities against critical inflammatory mediators, including NF-kB, COX-2, LOX enzyme, and inducible iNOS [22]. However, in the context of hepatotoxicity, little is known about the potential involvement of ERK/NF-kB signaling in regulating COX-2 expression and the possible COX-2-mediated production of pro-inflammatory cytokines in macrophages. Hence, in this context, it is prudent to investigate the relationship between signaling pathways like ERK/NF-kB activation and their impact on the potential COX-2-mediated hepatotoxicity induced by APAP, as well as the hepatoprotective effects of curcumin. In our study, following a single dose of APAP, there was a significant increase in the activity of ERK, NF-kB, and COX2, as indicated by their phosphorylation levels within the APAP group. However, in mice treated with curcumin, we observed a notable suppression of ERK and NF-kB protein phosphorylation, accompanied by a reduction in COX-2 expression. These findings collectively suggest that curcumin’s protective effects against APAP-induced hepatotoxicity in mice may be linked to its anti-inflammatory mechanisms, characterized by the attenuation of the ERK, NF-kB, and COX-2 signaling pathways. Building upon our results, we have demonstrated that curcumin effectively reduces liver enzyme levels and inflammation markers, while also diminishing liver neutrophils and macrophages in mice with APAP-induced liver injury. Curcumin administration notably alleviates APAP-induced liver injury by inhibiting the ERK, NF-kB, and COX-2 signaling pathways, consequently suppressing inflammatory responses. This alternative anti-inflammatory pathway, in conjunction with the previous antioxidant mechanisms, contributes to the overall hepatoprotective effects against APAP-induced hepatotoxicity. Our study offers a novel perspective by addressing the anti-inflammatory effect of curcumin on APAP-induced liver injury, shedding light on potential therapeutic strategies to mitigate liver damage.

While curcumin boasts impressive pharmacological potential, its clinical utility faces a multitude of challenges, notably stemming from low aqueous solubility, poor stability in body fluids‚ a high rate of metabolism, rapid clearance, diminished gastrointestinal absorption, and constrained bioavailability [53]. The limited bioavailability of curcumin directly influences its pharmacodynamic behavior. Hence, it is imperative that efforts are directed towards exploring novel drug-delivery systems for curcumin to unlock its complete clinical potential. In recent years, various strategies have emerged to bolster curcumin’s solubility and bioavailability, encompassing the use of curcumin analogues; the formation of chemical complexes with phospholipids, polysaccharides, or proteins; and the development of bioconjugates with substances such as turmeric oil or alanine [54]. The use of nanoformulations of curcumin and its derivatives has played a crucial role in advancing innovative treatment modalities [55]. However, it is vital to emphasize the critical necessity of first comprehending the molecular mechanisms through animal models before progressing to human applications. It remains imperative to emphasize the necessity for continued research efforts to gain a comprehensive understanding of the mechanisms underlying curcumin’s anti-inflammatory effects, particularly in the context of its potential therapeutic applications in humans, especially in conditions like APAP-induced liver injury. Curcumin’s promising anti-inflammatory properties show it to be a compelling candidate for ameliorating liver inflammation and affording protection against APAP-induced liver injury.

## 5. Conclusions

In summary, our study demonstrates that curcumin administration significantly alleviates APAP-induced liver damage. This favorable outcome is achieved through the inhibition of the ERK, NF-kB, and COX-2 signaling pathways, resulting in a reduction in downstream inflammatory responses. These findings indicate the potential of curcumin as a therapeutic candidate for improving APAP-induced hepatic injury, as evidenced by our experimental model. Nevertheless, further research is warranted to validate and broaden the clinical applications of curcumin in this context.

## Data Availability

The data presented in this study are available on request from the corresponding author.
